# Role of the ISKpn element in mediating mgrB gene mutations in ST11 hypervirulent colistin-resistant *Klebsiella pneumoniae*

**DOI:** 10.3389/fmicb.2023.1277320

**Published:** 2023-09-28

**Authors:** Lanlan Zhu, Ping Li, Guangyi Zhang, Zhiyong He, Xingyu Tao, Yicheng Ji, Wenjing Yang, Xiaofang Zhu, Wanying Luo, Wenjian Liao, Chuanhui Chen, Yang Liu, Wei Zhang

**Affiliations:** ^1^Department of Pulmonary and Critical Care Medicine, The First Affiliated Hospital of Nanchang University, Nanchang University, Nanchang, China; ^2^Jiangxi Institute of Respiratory Disease, The First Affiliated Hospital of Nanchang University, Nanchang, China; ^3^Yichun People’s Hospital, Yichun, China; ^4^First Clinical Medical College of Nanchang University, Nanchang University, Nanchang, China; ^5^Department of Hospital Infection Control, First Affiliated Hospital of Nanchang University, Nanchang University, Nanchang, China; ^6^National Regional Center for Respiratory Medicine, Jiang Xi Hospital of China-Japan Friendship Hospital, Nanchang, China; ^7^Department of Clinical Microbiology, The First Affiliated Hospital of Nanchang University, Nanchang University, Nanchang, China

**Keywords:** colistin-resistant *Klebsiella pneumoniae*, ISKpn, mgrB, ST11, hypervirulent

## Abstract

**Background:**

Colistin has emerged as a last-resort therapeutic against antibiotic-resistant bacterial infections, particularly those attributed to carbapenem-resistant Enterobacteriaceae (CRE) like CRKP. Yet, alarmingly, approximately 45% of multidrug-resistant *Klebsiella pneumoniae* strains now manifest resistance to colistin. Through our study, we discerned that the synergy between carbapenemase and IS elements amplifies resistance in *Klebsiella pneumoniae*, thereby narrowing the existing therapeutic avenues. This underscores the instrumental role of IS elements in enhancing colistin resistance through mgrB disruption.

**Methods:**

From 2021 to 2023, 127 colistin-resistant *Klebsiella pneumoniae* isolates underwent meticulous examination. We embarked on an exhaustive genetic probe, targeting genes associated with both plasmid-mediated mobile resistance-encompassing *bla*KPC, *bla*NDM, *bla*IMP, *bla*VIM, *bla*OXA-48-like, and *mcr-1* to *mcr-8*-and chromosome-mediated resistance systems, including *PhoP/Q*, *PmrA/B*, and *mgrB*. PCR amplification revealed the presence of virulence-associated genes from the *pLVPK* plasmid, such as *rmpA*, *rmpA2*, *iucA*, *iroB*, and *peg344*. *mgrB* sequencing was delegated to Sangon Biotech, Shanghai, and the sequences procured were validated using BLAST. Our search for IS elements was navigated through the IS finder portal. Phenotypically, we harnessed broth microdilution (BMD) to ascertain the MICs of colistin. To sketch the clonal lineage of mgrB-mutated CoR-Kp isolates, sophisticated methodologies like MLST and PFGE were deployed. S1-PFGE unraveled the intrinsic plasmids in these isolates. Our battery of virulence assessment techniques ranged from the string test and capsular serotyping to the serum killing assay and the *Galleria mellonella* larval infection model.

**Results:**

Among the 127 analyzed isolates, 20 showed an enlarged *mgrB* PCR amplicon compared to wild-type strains. These emerged over a three-year period: three in 2021, thirteen in 2022, and four in 2023. Antimicrobial susceptibility tests revealed that these isolates consistently resisted several drugs, notably TCC, TZP, CAZ, and COL. Additionally, 85% resisted both DOX and TOB. The MICs for colistin across these strains ranged between 16 to 64 mg/L, with a median of 40 mg/L. From a genetic perspective, MLST unanimously categorized these *mgrB*-mutated CoR-hvKp isolates as ST11. PFGE further delineated them into six distinct clusters, with clusters A and D being predominant. This distribution suggests potential horizontal and clonal genetic transmission. Intriguingly, every mgrB-mutated CoR-hvKP isolate possessed at least two virulence genes akin to the *pLVPK-like* virulence plasmid, with *iroB* and *rmpA2* standing out. Their virulence was empirically validated both *in vitro* and *in vivo*. A pivotal discovery was the identification of three distinct insertion sequence (IS) elements within or near the mgrB gene. These were:ISKpn26 in eleven isolates, mainly in cluster A, with various insertion sites including +74, +125, and an upstream −35.ISKpn14 in four isolates with insertions at +93, −35, and two upstream at −60.IS903B present in five isolates, marking positions like +74, +125, +116, and −35 in the promoter region. These diverse insertions, spanning six unique locations in or near the mgrB gene, underscore its remarkable adaptability.

**Conclusion:**

Our exploration spotlights the ISKpn element’s paramount role in fostering *mgrB* gene mutations in ST11 hypervirulent colistin-resistant *Klebsiella pneumoniae*. Employing MLST and PFGE, we unearthed two primary genetic conduits: clonal and horizontal. A striking observation was the ubiquitous presence of the *KPC* carbapenemase gene in all the evaluated ST11 hypervirulent colistin-resistant *Klebsiella pneumoniae* strains, with a majority also harboring the *NDM* gene. The myriad mgrB gene insertion locales accentuate its flexibility and the overarching influence of IS elements, notably the pervasive IS5-like variants ISKpn26 and IS903B. Our revelations illuminate the escalating role of IS elements in antibiotic resistance within ST11 hypervirulent colistin-resistant *Klebsiella pneumoniae*, advocating for innovative interventions to counteract these burgeoning resistance paradigms given their profound ramifications for prevailing treatment modalities.

## 1. Introduction

The dramatic rise in antibiotic resistance among Gram-negative bacteria since the 1970s has emerged as a significant global challenge (Nordmann and Poirel, 2019). Global resistance levels are alarmingly elevated, driven in part by the overuse and misuse of antibiotics and compounded by insufficient infection prevention and control strategies (Imai et al., 2019; Jean et al., 2022). Notably, strains of *Escherichia coli*, *Klebsiella pneumoniae* (KP), *Acinetobacter baumannii*, and *Pseudomonas aeruginosa*, presenting as multidrug-resistant (MDR), extensively drug-resistant (XDR), and pan-drug-resistant (PDR), are increasingly exhibiting diverse resistance mechanisms (Imai et al., 2019). As per data from the China Antimicrobial Surveillance Network, *Klebsiella pneumoniae* is distinguished as the second most epidemiologically pertinent pathogen within Chinese tertiary hospitals (Qiao et al., 2018). It is of particular concern that the incidence of carbapenem-resistant *Klebsiella pneumoniae* (CRKP) surged dramatically, from 3.0% in 2005 to 24.2% in 2022.^1^

*Klebsiella pneumoniae* (cKP) is a frequent cause of infections in healthcare settings, often harboring plasmids that code for antimicrobial resistance (Pu et al., 2023). In China, the sequence type (ST) 11 carbapenem-resistant *Klebsiella pneumoniae* (CRKP) stands as the predominant strain (Liao et al., 2020). The ST11 *Klebsiella pneumoniae*, renowned as the most widespread MDR lineage in Asia (Xu et al., 2017), was initially identified as a hypervirulent strain in China (Yang et al., 2022). While hypervirulent *Klebsiella pneumoniae* (hvKp) is acknowledged as a life-threatening pathogen, it has historically exhibited fewer associations with resistance compared to classical *Klebsiella pneumoniae* strains (Choby et al., 2020). HvKP is characteristically linked with invasive community-acquired infections and often carries virulence plasmids, like the *pLVPK* plasmid, which encodes key mucoid regulators such as rmpA, aerobactin, and salmochelin (Chen et al., 2004). Notably, the recent emergence of strains that are both carbapenem-resistant and hypervirulent (CR-HvKP) has led to exceedingly high mortality infections in various nations (Xie et al., 2021). Recent observations indicate that in Chinese intensive care units (ICUs), the ST11 hypervirulent CRKp strain (hv-CRKp) has been on the rise post-colonization by carbapenem-susceptible *Klebsiella pneumoniae* (CSKp) (Yang et al., 2022). This strain demonstrates heightened transmissibility, robust resistance, and significant virulence, posing a grave risk to public health (Jin et al., 2021; Xie et al., 2021).

In light of the multidrug resistance displayed by CRKP isolates, colistin, or polymyxin E, has been the mainstay therapeutic intervention for the most formidable antibiotic-resistant bacterial infections, especially those attributed to carbapenem-resistant *Enterobacteriaceae* (Falagas and Kasiakou, 2005). This antimicrobial agent exhibits efficacy against an array of Gram-negative bacilli (Zhang et al., 2018). By January 2017, China had approved polymyxin for intravenous therapeutic use in bacterial infection cases (Pan et al., 2018). Disturbingly, contemporary data indicate an approximate 45% colistin resistance rate among MDR *Klebsiella pneumoniae* (Falagas and Kasiakou, 2005). Capitalizing on the breakthroughs in whole-genome sequencing, Liu pinpointed the rise of colistin-resistant and hypervirulent MDR *Klebsiella pneumoniae* (CoR-HvKp) during 2017–2018 (Liu et al., 2022). In a parallel vein, Chen’s research highlighted the *in vivo* resistance emergence to colistin and tigecycline in carbapenem-resistant hypervirulent *Klebsiella pneumoniae* within China (Chen et al., 2021). Concerningly, strains resistant to polymyxin have made their presence felt on a global scale (Gharaibeh and Shatnawi, 2019).

Resistance to colistin in *Klebsiella pneumoniae* is primarily ascribed to mutations that cause dysregulation of the two-component systems (TCSs) *PmrAB*, *PhoPQ*, or *CrrAB* (Bhagirath et al., 2019). Additionally, the acquisition of a plasmid bearing the mobilized colistin resistance gene (mcr1) also confers resistance (Liu et al., 2016). Notably, while multiple TCSs contribute to lipid A modifications, mutations that nullify the functionality of the small regulatory protein *mgrB* account for colistin resistance in nearly 70% of *Klebsiella pneumoniae* strains (Jin et al., 2021; Bray et al., 2022). *mgrB* is a compact regulatory transmembrane protein, composed of 47 amino acids, that holds a pivotal function in antibiotic resistance (Bray et al., 2022). When inactivated, *mgrB* triggers an augmentation in lipid A modifications, subsequently conferring resistance to colistin (Groisman, 2001; Cheng et al., 2010). In the research spearheaded by Taher Uz Zaman et al., 23 non-replicating, colistin-resistant *Klebsiella pneumoniae* isolates were meticulously scrutinized. These isolates encompassed eight unique sequence types (STs) and predominantly manifested mutations in the *mgrB* or *PhoP* genes (Uz Zaman et al., 2018). Notably, ten isolates bore the ISKpn14 insertion sequence, while ISKpn28 and IS903 were identified in four and three isolates, respectively. It’s compelling to note the distinct strain distribution in their findings compared to our own, underscoring the innovative facet of our investigation. Parallelly, a study by Stephen Mark Edward Fordham et al. revealed that specific plasmids encode mobilizable IS elements. When integrated into the *mgrB* gene of *Klebsiella pneumoniae*, these elements lead to its inactivation, paving the way for colistin resistance (Fordham et al., 2022). The team’s exploration delved deep into evaluating the prevalence of *mgrB*-disruptive insertion sequences like ISL3 (ISKpn25), IS5 (ISKpn26), ISKpn14, and IS903B present on these plasmids. An intriguing find was the presence of antimicrobial resistance genes on these IS-rich plasmids, particularly those imparting resistance to carbapenems. Of these insertion sequences, ISKpn25 has garnered attention in multiple nations. In contrast, the prevalence of ISKpn26, ISKpn14, and IS903B appears to be acutely heightened in China. Under antibiotic stress, bacteria demand significant genomic modifications to introduce beneficial variations, which are beyond the scope of simple point mutations. Insertion sequences (ISs) play a pivotal role in enabling such changes. Many mutations in mgrB are mediated by these IS elements. In tandem with the presence of carbapenemase, these IS elements might amplify the drug resistance in *K. pneumoniae*, thereby constricting already limited treatment avenues. Expanding upon Bray et al.’s findings on the chromosome-mediated mechanism in CoR-HvKp isolates (Bray et al., 2022), our research accentuates the pronounced role of IS elements in facilitating colistin resistance through mgrB disruption.

## 2. Materials and methods

### 2.1. Bacterial isolates and clinical data collection

From May 2021 to April 2023, 127 distinct clinical colistin-resistant *Klebsiella pneumoniae* isolates were obtained from the First Affiliated Hospital of Nanchang University in China. Each isolate was identified using both the VITEK 2 automated system (bioMérieux, France) and the MALDI-TOF MS system (Bruker Daltonics, Billerica, MA, United States). All isolates were stored at −80°C until needed for further analysis. Antimicrobial susceptibility testing was conducted and interpreted according to the Clinical and Laboratory Standards Institute (CLSI) breakpoints (document M100-S32).

Clinical data for this study were sourced from the Electronic Medical Records of inpatients at the First Affiliated Hospital of Nanchang University. These data encompassed patient demographics, isolation dates, clinical diagnoses, specimen types, ward admissions, antimicrobial treatments, and hospitalization outcomes (Supplementary Table S2). The study’s methodologies and consent procedures received approval from the Ethical Committee of the First Affiliated Hospital of Nanchang University.

### 2.2. Identification of antibiotic-resistance and virulence-plasmid pLVPK-borne genes

All isolates were evaluated for the presence of genes coding for plasmid-mediated mobile-resistance genes (*bla*KPC, *bla*NDM, *bla*IMP, *bla*VIM, *bla*OXA-48-like, and mcr-1 to mcr-8), chromosome-mediated two-component systems (*PhoP/Q* and *PmrA/B*), *mgrB*, and virulence plasmid *pLVPK*-borne genes (*rmpA*, *rmpA2*, *iucA*, *iroN*, and *peg344*). This assessment was executed using polymerase chain reaction (PCR) amplification, in line with previously described methodologies (Chen et al., 2021). PCR products were then visualized through agarose gel electrophoresis.

For sequence analysis of *mgrB*, the services of Sangon Biotech (Shanghai, China) were utilized. Both nucleotide and the consequent protein sequences were subsequently analyzed using the Basic Local Alignment Search Tool (blaST) program available at the National Center for Biotechnology Information website.^2^ Insertion sequences (ISs) were evaluated via the IS finder website.^3^

Of note, 20 isolates yielded an *mgrB* PCR amplicon product noticeably larger than that observed in wild-type strains (Supplementary Figure S1). To elucidate the cause of this amplified gene size, these 20 mutated *mgrB* CoR-Kp isolates were selected for further analysis (Poirel et al., 2015).

### 2.3. Colistin antimicrobial susceptibility testing

The minimum inhibitory concentrations (MICs) of colistin were ascertained using the broth microdilution (BMD) method, recognized as the gold-standard reference in line with the Clinical and Laboratory Standards Institute (CLSI) M100-S32 criteria. With the standard transitioning from resistance breakpoints to an intermediate categorization, susceptibility evaluations for colistin were conducted following the guidelines established by the European Committee on Antimicrobial Susceptibility Testing (EUCAST). For *Klebsiella pneumoniae*, colistin MICs were interpreted as follows: susceptibility is denoted by MIC values ≤2 mg/L, while resistance is indicated by values >2 mg/L.

### 2.4. Molecular analyses

#### 2.4.1. Multilocus sequence typing and pulsed-field gel electrophoresis

MLST and PFGE were used for assessing the clonal relationship of the selected *mgrB*-mutated CoR-Kp isolates.

MLST employs seven conserved housekeeping genes: *gapA*, *infB*, *mdh*, *pgi*, *phoE*, *rpoB*, and *tonB*, as per the guidelines provided on the Pasteur Institute MLST website. The resulting amplicons were purified and dispatched to Sangon Biotech (Shanghai, China) for sequencing. The resultant sequences were then matched with those catalogued in the MLST database to establish the sequence type (ST) (Li et al., 2023).

PFGE was executed following the standardized protocol recommended by the CDC (CDC, 2013). The enzyme XbaI from TaKaRa was employed for the procedure. The generated DNA fragments were segregated using the CHEF DR III system (Bio-Rad, Richmond, CA, United States). To serve as a molecular weight standard, the *Salmonella serotype Braenderup* strain H9812 was incorporated. Following the electrophoretic separation, the generated DNA patterns were then analysed using the BioNumerics software (version 7.6). The software facilitated the construction of a dendrogram based on the unweighted Pair-Group Method with Arithmetic means (UPGMA) and employed the Dice similarity coefficient (SD). The setting allowed for a 1.5% position tolerance. For the isolates to be deemed genetically similar, it was essential for their Dice coefficient correlation to surpass 80%. This threshold was set based on the “possibly related (4–6 bands difference)” criteria as posited by Tenover et al. (1995).

#### 2.4.2. S1-pulsed field gel electrophoresis

S1-PFGE, utilizing S1 nuclease from TaKaRa, was employed to evaluate the plasmid content of *mgrB*-mutated CoR-Kp. The separation of plasmid fragments was accomplished using the CHEF DR III system (Bio-Rad, Richmond, CA, United States), with the *Salmonella serotype Braenderup* isolate H9812 as a molecular reference.

### 2.5. Virulence assessment of *mgrB*-mutated CoR-Kp

#### 2.5.1. Hyperviscous phenotype detection (string test)

The hypermucoviscous phenotype was assessed using the string test. A positive result was defined by the formation of strings ≥5 mm upon stretching with a sterile inoculation loop. For this test, *Klebsiella pneumoniae* strains NTUH-K2044 and ATCC700603 served as positive and negative controls, respectively.

#### 2.5.2. Serum killing assay

A serum killing assay was conducted to evaluate *in vitro* virulence, adapted from the method previously described (Li et al., 2023). Serum was procured from healthy donors and preserved at −80°C. Bacterial inocula, at a concentration of 106 CFU during the mid-log phase, were exposed to 75% pooled human serum. The enumeration of viable bacteria was conducted at intervals of 0, 1, 2, and 3 h post-exposure, under conditions of 37°C and 200 rpm agitation. Each bacterial strain underwent a minimum of three independent assays. The serum susceptibility was delineated into six gradations, which were further categorized as: highly sensitive (grades 1–2), intermediately sensitive (grades 3–4), resistant (grades 5–6). Grade 1 designation implied that the viable count was <10% of the original inoculum post 1 and 2 h, and diminished to <0.1% at the 3 h mark. In contrast, grade 2 was characterized by a viable count between 10 and 100% post the 1 h interval but was less than 10% after 3 h. Grade 3 counts surpassed the initial inoculum after 1 h but remained below 100% at the subsequent 2 h and 3 h intervals. Grade 4 counts consistently exceeded the original inoculum after 1 and 2 h, but fell short of 100% after 3 h. Grade 5 counts continuously surpassed the inoculum across all time intervals, but evidenced a decrement in the third hour. Lastly, a grade 6 classification was characterized by consistently increasing viable counts across all time points. *Klebsiella pneumoniae* ATCC 700603 and hvKP strain NTUH-K2044 were utilized as benchmark controls, with serum sensitivities graded at 2 (sensitive) and 5 (resistant), respectively.

#### 2.5.3. *Galleria mellonella* infection model

*In vivo* virulence was assessed using the *Galleria mellonella* infection model, a technique previously established (Tsai et al., 2016). Specifically, 10 pathogen-free *Galleria mellonella* larvae, each weighing between 250 and 350 mg (sourced from Tianjin Huiyude Biotech Company, Tianjin, China), were selected for each bacterial strain. Mid-log-phase cultures were prepared, washed, and resuspended in PBS. Each larva was then inoculated with 1 × 10^6 CFU in a 10 uLvolume, delivered to the hemocoel through the rear left proleg. Larval survival was monitored at 24 h intervals over a span of 4 days, maintaining the larvae in a dark environment at 37°C within petri dishes. These tests were replicated thrice. The LD50 value, derived from the Galleria model, serves as a marker for determining hypervirulence in *Klebsiella pneumoniae* isolates. For reference controls, the HvKP strain NTUH-K2044 represented high virulence, while PBS denoted low virulence.

## 3. Results

### 3.1. Overall prevalence of colistin-resistant *Klebsiella pneumoniae* strains in a Chinese tertiary hospital

**Figure 1:**
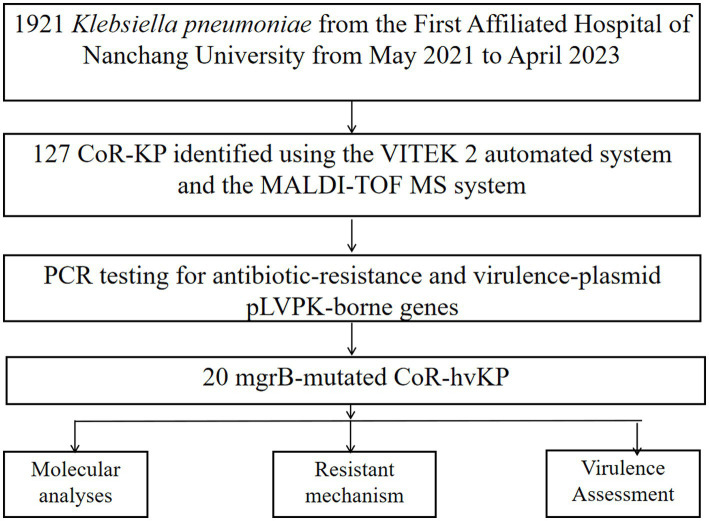
Flowchart illustrating the step-by-step methodology employed in the study.

Between May 2021 and April 2023, our hospital collected 1,921 clinical isolates of *Klebsiella pneumoniae*. Out of these, 127 (6.6%) were identified as colistin-resistant strains. Further screening via PCR amplification and sequencing identified 20 of these colistin-resistant strains as having mutations in the *mgrB* gene (Figure 1; Supplementary Figure S1).

### 3.2. Antimicrobial susceptibility testing for *mgrB* mutation colistin-resistant *Klebsiella pneumoniae* isolates

**Figure 2:**
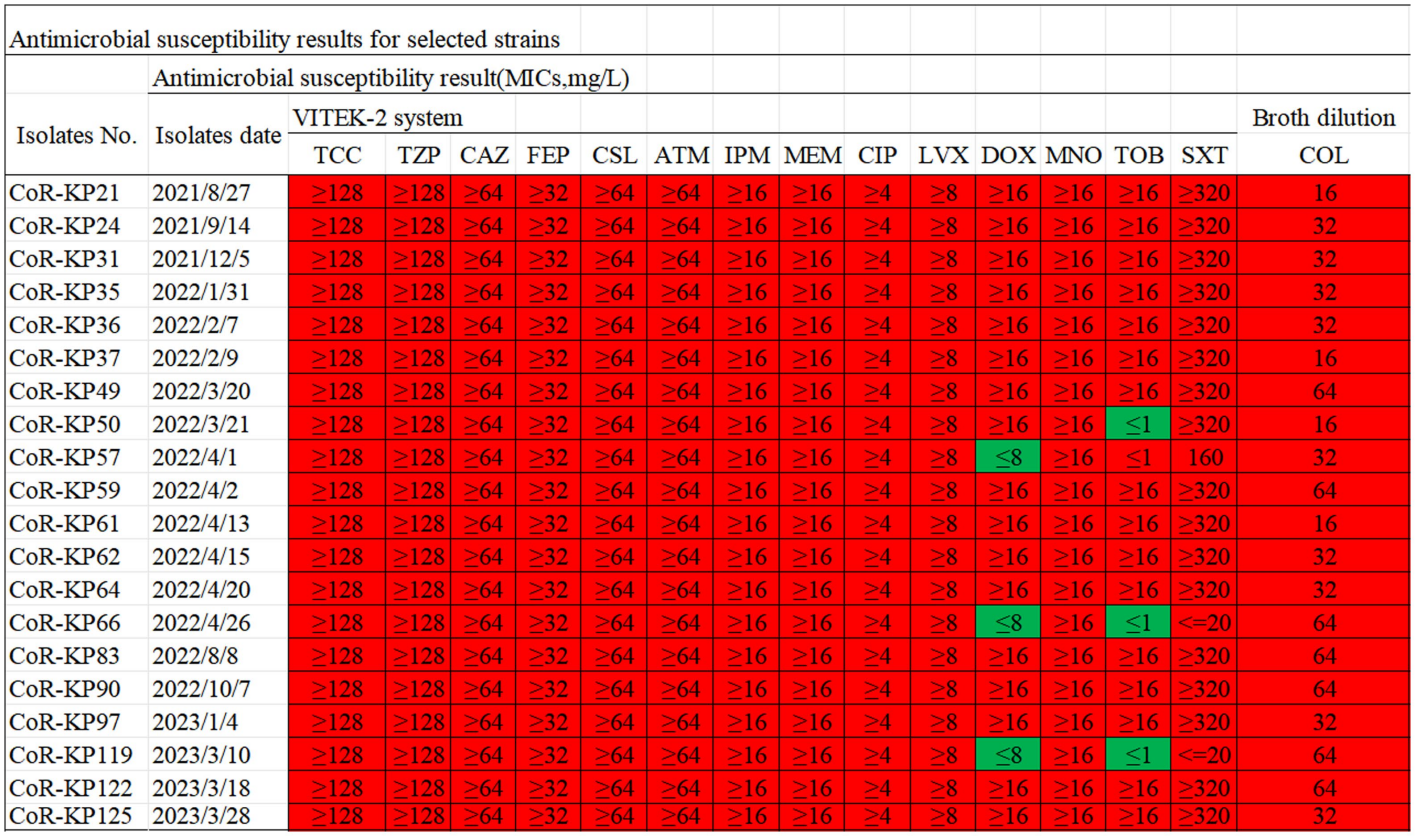
Antibiotic susceptibility profiles for the 20 *mgrB*-mutated CoR-Kp isolates. TCC, Ticarcillin; TZP, Piperacillin/Tazobactam; CAZ, Ceftazidime; FEP, Cefepime; CSL, Cefoperazone/Sulbactam; ATM, Aztreonam; IPM, imipenem; MEM, meropenem; CIP, Ciprofloxacin; LVX, Levofloxacin; DOX, Doxycycline; MNO, Minocycline; TOB, Tobramycin; SXT, Trimethoprim/Sulfamethoxazole; COL, colistin; Red, resistant; Green, susceptible.

Antimicrobial susceptibility testing of the 20 *Klebsiella pneumoniae* isolates with *mgrB* mutations revealed universal resistance to TCC, TZP, CAZ, FEP, CSl, ATM, CIP, LVX, MNO, SXT, IPM, MEM, and COL. Additionally, 85% of the strains exhibited resistance to DOX and TOB (Figure 2). The colistin MIC values for these strains spanned a range of 16–64 μg/mL, with a median value of 40 μg/mL (Figure 2).

### 3.3. Demographic characteristics and molecular typing of *mgrB* mutation hypervirulent colistin-resistant *Klebsiella pneumoniae* isolates

As detailed in Supplementary Table S1, the temporal distribution of isolates was as follows: 3 in 2021, 13 in 2022, and 4 in 2023. The patients from whom these isolates were collected had a median age of 58.8 ± 14.7 years, spanning from 23 to 83 years. Males represented 55% (11/20) of the patients. The isolates originated from diverse clinical sources, such as sputum, blood, bronchoalveolar lavage fluid (BALF), pus and others. These patients were admitted across various hospital wards, including the intensive care unit (ICU), respiratory department, neurosurgery department, hematology department, gastroenterology department, rehabilitation department, and other specialized wards. Notably, half of the patients (10/20) were in the ICU. The observed mortality rate amongst this cohort was 60% (12/20), as depicted in Supplementary Table S1.

**Figure 3:**
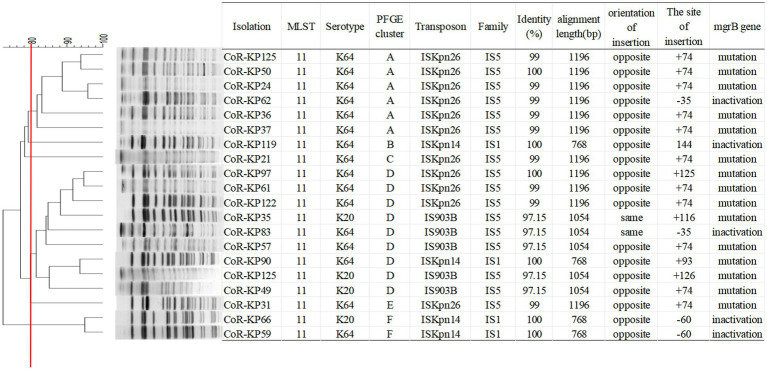
PFGE typing of the 20 *mgrB*-mutated CoR-Kp isolates. Genomic DNA from each research strain was digested with Xba I. The digests were then subjected to PFGE, producing diagnostic genomic DNA fragmentation fingerprints. The dendrogram representing the PFGE profiles was clustered using UPGAMA based on Dice similarity, analyzed with the bionumerics software. The red line marks the 80% similarity boundary.

As highlighted in Figure 3, MLST analysis revealed that all *mgrB*-mutated CoR-hvKp isolates were categorized as ST11 (100%, 20/20). PFGE analysis discerned six distinct clusters among the 20 isolates, with the majority clustering within groups A and D. This clustering suggests dual genetic transmission modes for these strains: both horizontal and clonal transmission.

**Figure 4:**
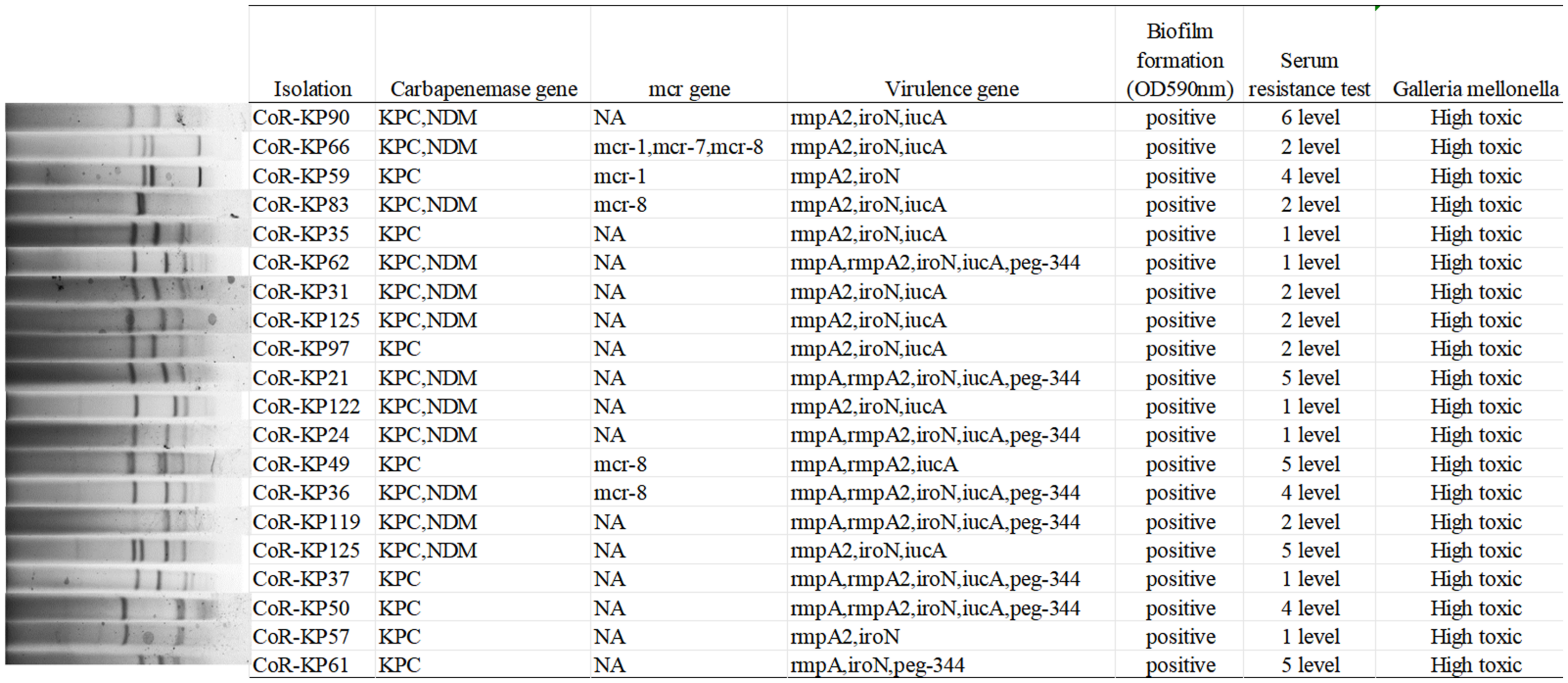
S1-PFGE typing of the 20 *mgrB*-mutated CoR-Kp isolates. Genomic DNA from each research strain was digested with S1, and the resultant DNA fragments were separated using a CHEF DR III apparatus.

As depicted in Figure 4, the primary carbapenemase determinants were *bla*KPC (100%, 20/20) and *bla*NDM (60%, 12/20). None of the isolates tested positive for the OXA-48, VIM, or IMP genes. Supplementary Table S1 provides a comprehensive breakdown of the distribution of resistance genes, virulence factors, and sequence types (STs) among the isolates.

All isolates carried the pLVPK virulence plasmid, with distributions as follows: *rmpA* (45%, 9/20), *rmpA2* (95%, 19/20), *iroN* (95%, 19/20), *iucA* (85%, 17/20), and *peg344* (40%, 8/20). Notably, none of the isolates tested positive for the *PhoQ/PhoP* and *pmrA/B* genes. Furthermore, five isolates harbored both the mutated *mgrB* gene and a mcr positive gene, specifically *mcr-1* (2/20), *mcr-7* (1/20), and *mcr-8* (4/20).

### 3.4. Virulence analysis of *mgrB* mutation hypervirulent colistin-resistant *Klebsiella pneumoniae* isolates

As illustrated in Figure 4, all the *mgrB* mutative CoR-hvKP isolates were found to harbor at least two virulence genes situated on a *pLVPK*-like virulence plasmid, including the *iroN*, *iucA*, *peg-344*, *rmpA*, and *rmpA2* genes. Both the string test and biofilm formation assays yielded positive results for these isolates, signifying their virulence potential. Capsular serotyping identified 16 isolates as K64 and 4 as K20, with no detection of K1 and K2 types (Figure 3). Interestingly, the K64 serotype is noted as the most prevalent among *KPC*-2-producing *Klebsiella pneumoniae* in China.

These results underline the isolates’ hypervirulent nature, evidenced by the presence of hypermucoviscosity and plasmid-borne genes akin to *pLVPK*. *In vitro* assessments corroborated this observation, with the strains displaying marked serum resistance; the survival rate was approximately 95% following a 60 min incubation with serum (Figure 4).

Further validation of the hypervirulent phenotype in all these CoR-hvKP was obtained through the *Galleria mellonella* infection model. As depicted in Figure 4, when a 10^6 CFU suspension of the isolates was used to infect *Galleria mellonella* larvae, all isolates exhibited a survival rate of fewer than 48 h. This pattern was akin to the virulent strain NTUH-K2044, emphasizing the notable virulence attributes of these CoR-hvKP isolates.

### 3.5. Iskpn element as a key target for inactivation/mutation of the *mgrB* in CoR-HvKp

**Figure 5:**
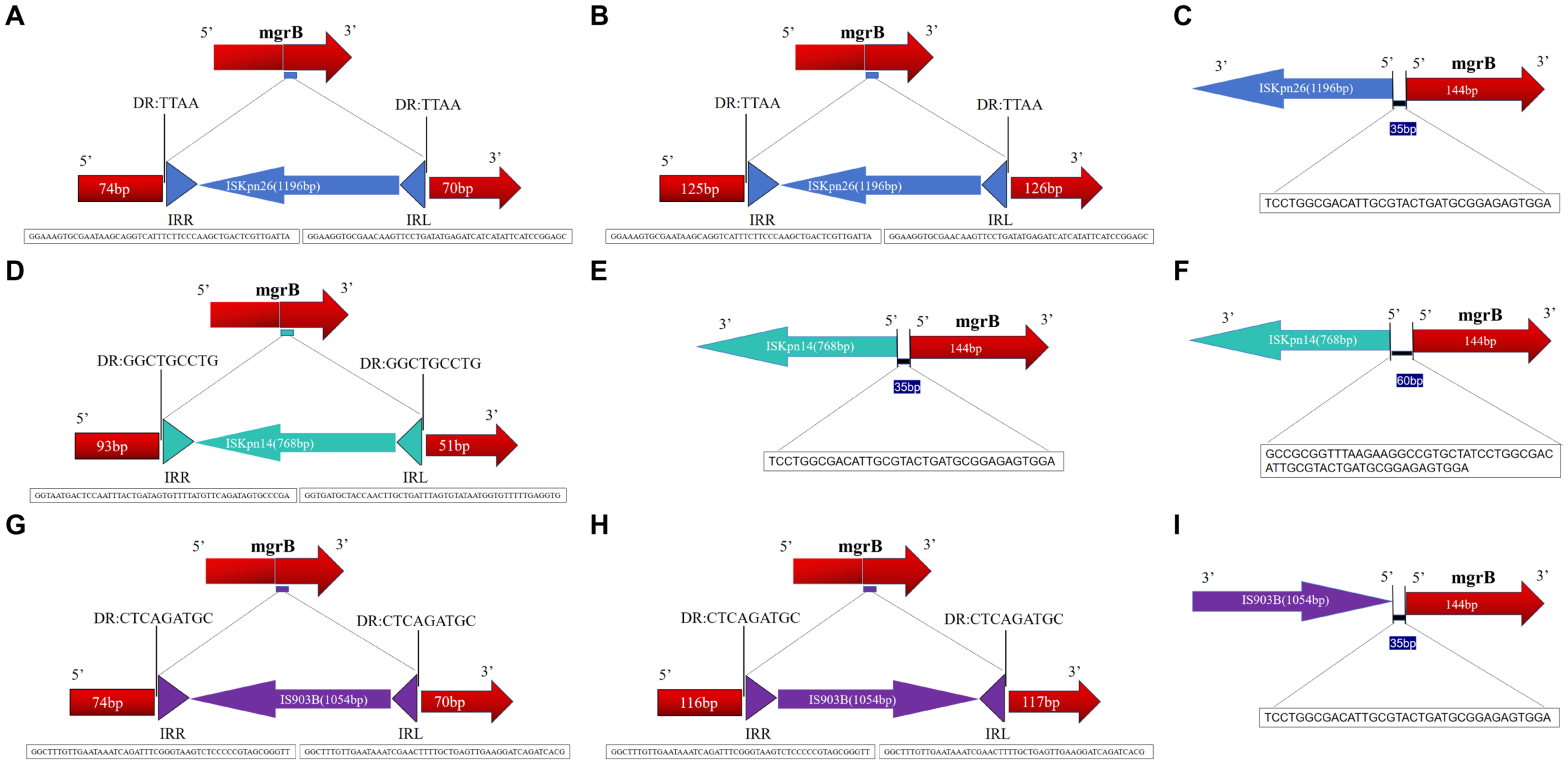
Schematic depiction of distinct insertion events observed within the *mgrB* gene. The left and right inverted repeats corresponding to each IS are denoted as IRR and IRL, respectively. (1) For the 11 isolates featuring the ISKpn26 insertion: nine exhibited insertions at position +74 **(A)**; one had an insertion at +125 **(B)**; one displayed an insertion at −35, located in the promoter region upstream of the start codon **(C)**. (2) Among the four isolates with the ISKpn14 insertion: one demonstrated an insertion at +93 **(D)**; one had its insertion at −35 **(E)**; two revealed insertions at −60, situated in the promoter region upstream of the start codon **(F)**. (3) For the five isolates bearing the IS903B insertion: two presented insertions at +74 **(G)**; one showed insertions each at positions +125 **(H)**, +116 **(I)**, and −35 (located in the promoter region upstream of the start codon).

The *mgrB* gene was PCR-amplified in 127 CoR-Kp isolates. Subsequent sequence analysis revealed that 20 isolates produced a larger amplicon relative to a wild-type isolate (Supplementary Figure S1). Three distinct elements from two IS families were identified, either within the open reading frame (ORF) or the promoter of the *mgrB* gene. Of the 127 isolates, 11 carried ISKpn26, four harbored ISKpn14, and five contained IS903B within their *mgrB* gene. The orientation and insertion site varied, as illustrated in Figure 5.

In the eleven isolates with ISKpn26, nine had insertions at position +74, one at +125, and one at −35 (promoter region, upstream of the start codon) (Figures 5A–C). Among the four isolates with ISKpn14, one showed insertion at +93, one at −35, and two at −60 (promoter region, upstream of the start codon) (Figures 5D–F). Of the five isolates with IS903B, two had insertions at +74, one each at +125, +116, and −35 (promoter region, upstream of the start codon) (Figures 5G–I). The left and right inverted repeats (IRs) were delineated, with the sequences as follows: ISKpn26 IRL: GGAAGGTGCGAACAAGTTCCTGATATGAGATCATCATATTCATCCGGAGC, IRR: GGAAAGTGCGAATAAGCAGGTCATTTCTTCCCAAGCTGACTCGTTGATTA; ISKpn14 IRL: GGTGATGCTACCAACTTGCTGATTTAGTGTATAATGGTGTTTTTGAGGTG, IRR: GGTAATGACTCCAATTTACTGATAGTGTTTTATGTTCAGATAGTGCCCGA; IS903B IRL: GGCTTTGTTGAATAAATCGAACTTTTGCTGAGTTGAAGGATCAGATCACG, IRR: GGCTTTGTTGAATAAATCAGATTTCGGGTAAGTCTCCCCCGTAGCGGGTT (Figures 5A,D,G). The direct repeat (DR) sequences were: ISKpn26 DR: TTAA; ISKpn14 DR: GGCTGCCTG; IS903B DR: CTCAGATGC (Figures 5A,D,G). Further details on transposons identified in the 20 mgrB-mutated CoR-HvKp isolates using ISfinder are provided in Supplementary Table S3. No mutations were detected in other components of the signaling system, such as the *PhoP/Q* and *PmrA/B* compartments.

## 4. Discussion

Colistin and tigecycline represent some of the limited therapeutic options available for infections caused by carbapenem-resistant Enterobacteriaceae (CRE) (Gharaibeh and Shatnawi, 2019). Alarmingly, the prevalence of carbapenem-resistant hypervirulent *Klebsiella pneumoniae* (CR-hvKP) in China is higher than previously anticipated (Yang et al., 2022). This increased prevalence raises concerns about the potential acquisition of resistance to colistin, tigecycline, and ceftazidime/avibactam in CR-hvKP, potentially leading to severe clinical consequences (Liao et al., 2020). To counteract this emerging trend, it is imperative to manage and judiciously apply antibiotics.

In this study, we provide insights into the molecular mechanisms governing colistin resistance in ST11 hypervirulent *Klebsiella pneumoniae*. Utilizing MLST and PFGE analyses, we discerned two predominant genetic transmission modes among these strains: clonal and horizontal. Intriguingly, among the *mgrB*-mutated CoR-HvKp isolates, five tested positive for the plasmid-borne mcr gene. This finding underscores the convergence of both chromosomal and plasmid-mediated resistance strategies. Our observations are consistent with the recent work of Zhang et al. (2018). A salient point from our findings is that, in comparison to *mgrB* mutations (75%, 15/20), the inactivation of *mgrB* (25%, 5/20) manifested a pronounced increase in colistin resistance, in tandem with augmented virulence.

Previous literature has established that the *mgrB* gene alterations are prevalent mechanisms attributing to colistin resistance in *Klebsiella pneumoniae* (Zhang et al., 2018; Bray et al., 2022). In a comprehensive study by Stephen and colleagues, it was elucidated that certain plasmids encode for mobilizable IS elements. These elements, when integrated into the *mgrB* gene of *Klebsiella pneumoniae*, result in the gene’s inactivation, thereby leading to colistin resistance. The research delved into assessing the prevalence of specific *mgrB*-gene disruptive insertion elements, namely ISL3 (ISKpn25), IS5 (ISKpn26), ISKpn14, and IS903B, present on these plasmids. Additionally, these plasmids containing IS elements underwent an extensive analysis to identify antimicrobial resistance genes, with a keen focus on those that confer resistance to carbapenems. Notably, while ISKpn25 is widespread and found in numerous countries, the occurrences of ISKpn26, ISKpn14, and IS903B are particularly pronounced in China (Fordham et al., 2022). The IS5-like insertion element is predominantly implicated in *mgrB* disruption in this bacterium (Poirel et al., 2015). In our cohort, we discerned that 80% of the IS elements (namely ISKpn26 and IS903B) are members of the IS5 family, while the remaining 20% (ISKpn14) affiliate with the IS1 family (Supplementary Table S2). It’s worth noting that ISKpn26 displays 99% amino acid similarity to IS5, but this similarity diminishes to 91% at the DNA level. IS903B, another member of the IS5 family, diverges from both IS903 and IS102 by 34 and 61 nucleotides, respectively. Historically, IS903, an IS5 family member, has been implicated in antibiotic resistance, functioning either as a resistance gene carrier or as an agent modifying antibiotic targets.

Among the isolates, eleven with ISKpn26 insertion were grouped into four distinct PFGE clusters (A, C, D, E). Notably, all isolates in the PFGE cluster A exhibited ISKpn26 insertion. Meanwhile, the four isolates with ISKpn14 insertion were categorized into three PFGE clusters (B, D, F). These findings suggest that ISKpn26 and ISKpn14 potentially employ dual genetic transmission modes: horizontal and clonal. Contrarily, IS903B may represent a single clonal expansion, as all five isolates with this insertion aligned with the single PFGE cluster D.

The mutations in mgrB were mostly mediated by insertion elements (IS). Interestingly, isolates carrying either of the two insertion elements (IS903B and ISKpn14) were found to harbor more mcr genes. This finding indicates that the colistin resistance mechanism in these strains (IS903B and ISKpn14) involves both chromosomal and plasmid-mediated factors. In a parallel study conducted by Taher Uz Zaman and his team, 23 non-replicating colistin-resistant *Klebsiella pneumoniae* isolates from Europe were investigated. These isolates spanned eight distinct sequence types (STs) and exhibited mutations primarily in either the *mgrB* or *PhoP* genes. Notably, ISKpn14 was detected in 10 of the isolates, ISKpn28 in four, and IS903 in three. A striking observation was the different strain distribution when compared to our findings, highlighting the innovative aspect of our research (Taher Uz Zaman et al., 2018). The orientation and insertion sites exhibited variability, as demonstrated in Figure 5. Despite the IS element being inserted across seven distinct locations within the *mgrB* gene, the +74 site emerges as a preferential insertion hotspot. Furthermore, 11 isolates from the IS-5 family (ISKpn26 and IS903B) shared the same integration site (+74) within the *mgrB* gene but fell into disparate PFGE clusters. This pattern insinuates a probable recombination event at this chromosomal location. Further investigations are warranted to explore the spacers potentially triggering the translocation of these elements from plasmids to the chromosome, a shift that could metamorphose the multi-drug-resistant pathogen into a pan-drug-resistant entity.

The key discovery of this study is the prevalent role of the ISKpn element in the inactivation or mutation of the *mgrB* gene in ST11 hypervirulent colistin-resistant *Klebsiella pneumoniae*. Remarkably, all the isolates in this study exhibited hypervirulent characteristics. Our data reveals that ST11 Hypervirulent *Klebsiella pneumoniae* is the most common strain circulating in our hospital, yet the isolates carrying the IS element in this study date from 2021, pinpointing the insertion as a recent phenomenon. Hypervirulent colistin-resistant *Klebsiella pneumoniae* with *mgrB* mutations have recently been reported in various regions globally, signaling the emergence of an endemic ColR clone (Dong et al., 2018; Bolourchi et al., 2021; Chen et al., 2021; Liu et al., 2022).

Another pivotal observation from our study is the pronounced prevalence of carbapenemase genes, notably *bla*KPC (present in 100%, 20/20 of the samples) and *bla*NDM (in 55%, 11/20), within the ST11 hypervirulent colistin-resistant *Klebsiella pneumoniae*. This suggests the IS element might have a contributory role in mediating resistance, extending beyond colistin to encompass carbapenems. This aligns with prior research on tigecycline resistance in *Klebsiella pneumoniae*, where the IS5 element is integrated into the promoter region of the putative efflux pump operon, kpgABC (Schnetz and Rak, 1992; Zhang and Saier, 2009). There’s a pressing need for further studies to elucidate the mechanisms-specifically the spacers-prompting these elements’ transfer from plasmids to the chromosome. Such a shift has the potential to escalate a multi-drug-resistant pathogen to a pan-drug-resistant phenotype (Lv et al., 2020; Sun et al., 2020).

Single nucleotide deletions leading to frame shifts and subsequent premature stop codons have been extensively documented in colistin resistance among *Klebsiella pneumoniae* and other bacterial species (Dong et al., 2018; Lv et al., 2020). In our study, we observed disruptions in the *mgrB* gene by IS elements. Such disruptions shortened the CDS region of the *mgrB* gene to less than 144 bases, causing a frame shift. Despite the limited number of isolates examined, our findings resonate with the hypothesis that, under stressors like antibiotic exposure, bacteria may necessitate significant genomic structural modifications to introduce vital adaptive variations. Such modifications could be beyond the reach of mere point mutations, making IS elements a more effective mechanism for these changes (Schnetz and Rak, 1992; Poirel et al., 2015; Dong et al., 2018).

## 5. Conclusion

Our research highlights the critical role of the ISKpn element in the mutation of the *mgrB* gene in ST11 hypervirulent colistin-resistant *Klebsiella pneumoniae*. Through MLST and PFGE analyses, we delineated two principal genetic transmission methods, clonal and horizontal. Notably, all the studied isolates harbored the *bla*KPC carbapenemase gene, while 55% also presented the *bla*NDM gene. The extensive insertion points observed within the *mgrB* gene exemplify its inherent adaptability. Particularly dominant were the IS5-like elements, ISKpn26 and IS903B. These findings illuminate the increasing significance of IS elements in antibiotic resistance, especially via *mgrB* disruption. The adaptability of the *mgrB* gene underscores bacterial evolutionary dynamics, emphasizing the pressing need for advanced strategies to address these evolving resistance mechanisms, given the challenges they introduce to existing therapeutic approaches.

## Data availability statement

The original contributions presented in the study are included in the article/Supplementary material, further inquiries can be directed to the corresponding authors.

## Author contributions

LZ: Investigation, Methodology, Writing – original draft. PL: Investigation, Methodology, Writing – original draft. GZ: Formal analysis, Writing – original draft. ZH: Formal analysis, Writing – original draft. XT: Formal analysis, Writing – original draft. YJ: Writing – original draft. WY: Writing – original draft. XZ: Writing – original draft. LW: Formal analysis, Writing – original draft. WL: Writing – original draft. CC: Writing – original draft. YL: Conceptualization, Writing – review & editing. WZ: Conceptualization, Writing – review & editing.

## Footnotes

^1^ https://www.chinets.com/

^2^www.ncbi.nlm. nih.gov

^3^ www-is.biotoul.fr
