# Supplementary material for: Role of the ISKpn element in mediating mgrB gene mutations in ST11 hypervirulent colistin-resistant Klebsiella pneumoniae
Source: Front Microbiol. 2023 Sep 28;14:1277320. doi: 10.3389/fmicb.2023.1277320 (PMC10569121; doi:10.3389/fmicb.2023.1277320)

Supplementary figure

Figure legend: Gel electrophoresis of PCR products targeting mgrB. The DNA gel displays PCR products with their expected lengths. Lane M: 2000 bp size marker. The expected size for the mgrB product is approximately 380 bp. Among the samples, 20 isolates produced a mgrB PCR amplicon noticeably larger than that of the wild-type (WT) strain. Note: No amplification was observed in the negative control (water control, C−).

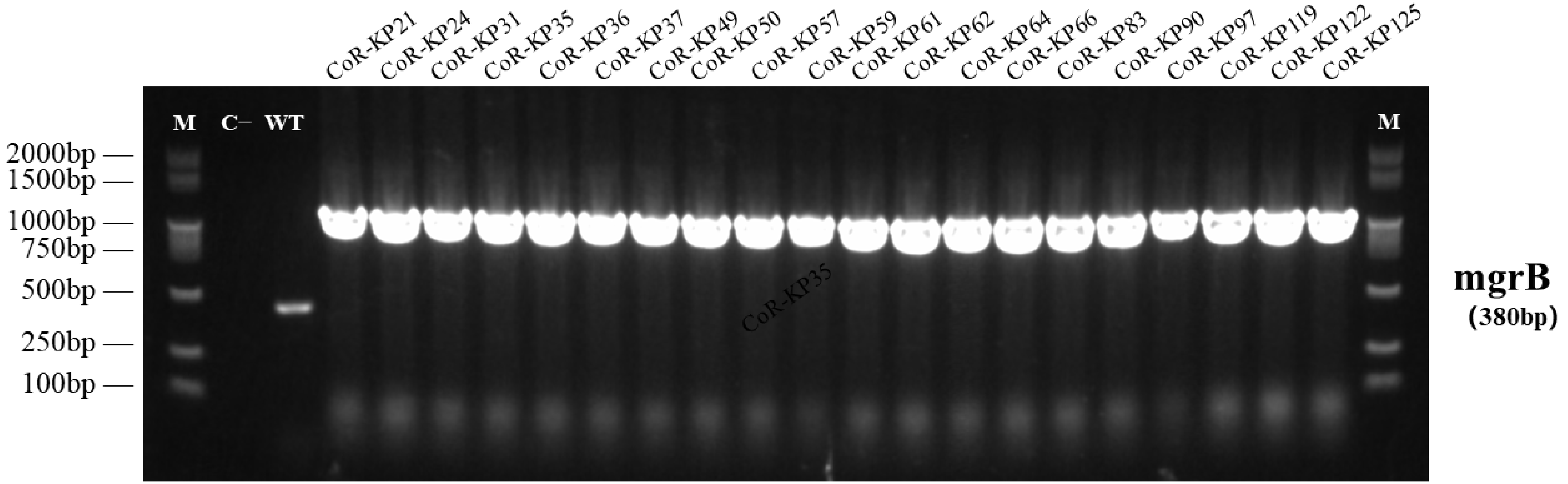

Supplement: Supplementary file 4 [file Image_1.pdf]
